# Multitasking behaviors and provider outcomes in emergency department physicians: two consecutive, observational and multi-source studies

**DOI:** 10.1186/s13049-020-00824-8

**Published:** 2021-01-07

**Authors:** Tobias Augenstein, Anna Schneider, Markus Wehler, Matthias Weigl

**Affiliations:** 1 Institute and Clinic for Occupational, Social and Environmental Medicine, University Hospital, LMU Munich, Ziemssenstrasse 1, 80336 Munich, Germany; 2 Department of Emergency Medicine and Department of General, Visceral and Trauma Surgery, Academic Hospital Porz am Rhein, Urbacher Weg 19, 51149 Cologne, Germany; 3 Institute of Medical Sociology and Rehabilitation Science, Charité – Universitaetsmedizin Berlin, Charitéplatz 1, 10117 Berlin, Germany; 4 Department of Emergency Medicine and Department of Medicine IV, University Hospital Augsburg, Stenglinstrasse 2, 86156 Augsburg, Germany; 5 Institute for Patient Safety, University Hospital Bonn, Bonn, Germany

**Keywords:** Emergency medicine, Physicians, Clinical care, Multitasking, Cognitive systems, Work stress, Situation awareness, Observation

## Abstract

**Background:**

Multitasking is a key skill for emergency department (ED) providers. Yet, potentially beneficial or debilitating effects for provider functioning and cognition are underexplored. We therefore aimed to investigate the role of multitasking for ED physicians’ work stress and situation awareness (SA).

**Methods:**

Two consecutive, multi-source studies utilizing standardized expert observations in combination with physicians’ self-reports on stress and SA were set out in an academic ED. To control for ED workload, measures of patient acuity, patient counts, and ED staff on duty were included. Regression analyses estimated associations between observed proportion of time spent in multitasking with matched ED physicians’ reports on stress (study 1) and SA (study 2).

**Results:**

ED physicians engaged between 18.7% (study 1) and 13.0% (study 2) of their worktime in multitasking. Self-reported as well as expert-observed multitasking were significantly associated. This confirms the internal validity of our observational approach. After controlling for ED workload, we found that physicians who engaged more frequently in multitasking perceived higher work stress (Beta = .02, 95%CI .001–.03; *p* = .01). In study 2, ED physicians with more frequent multitasking behaviors reported higher SA (B = .08, 95%CI .02–.14; *p* = .009).

**Conclusions:**

Multitasking is often unavoidable in ED care. Our findings suggest that ED physicians’ multitasking increases stress experiences, yet, may facilitate professional’s experiences of situation awareness. Our results warrant further investigation into potentially ambivalent effects of ED providers’ multitasking in effectively sharing time between competing demands while maintaining performance and safety.

## Background

Due to the very intricacies of emergency care work, ED physicians often face competing care demands with needs to manage multiple events concurrently. ED physicians tend to apply multitasking when multiple events load upon them within often resource-limited and interrupt-driven workflows [1–4]. We define multitasking as simultaneous task performance, i.e., when physicians combine concurrent activities, e.g., completing a patient chart and synchronously answering a phone call [3, 5]. ED physicians perform multitasking during a substantial amount of their work time with previously observed shares between 10.7% up to 30.6% [6–9].

ED providers engage in multitasking to maintain fast and efficient care in time-constrained ED environments, e.g., keeping several patient charts at hand or open (i.e., open active EMR charts) to facilitate access and maintain fast response [3, 10, 11]. While juggling with competing priorities, ED physicians apply multitasking as a prompt-response to disruptive care demands to adjust communication and care strategies [4, 12]. Therefore, it has been proposed that multitasking is a key ability of ED providers [3, 13–15]. Although ED physicians frequently operate under a barrage of multiple and competing demands, the very consequences of multitasking for provider outcomes are not well understood [3, 10, 11, 16]. Particularly the following questions remain unresolved:

First, there is a paucity of naturalistic studies on multitasking demands in acute care settings with real observational data that closely approximate ED conditions under which multitasking takes place [10, 16, 17]. Secondly, a thorough understanding concerning multitasking and its consequences for provider outcomes such as work stress and cognition is needed [3]. ED physicians’ mental resources play a vital role in achieving multiple clinical tasks over time and delivering safe care [18]. Yet, naturalistic studies that quantify physician cognition in complex multi-patient environments are lacking [19]. Situation awareness is a key challenge in dynamic and information-rich EDs where providers need to maintain alertness to safeguard provision of optimal patient care as well as frequently reconcile care resources with patient care demands [20–22]. We hypothesize that multitasking impairs provider’s situation awareness due to increased mental load while working with multiple patients in team-based care settings with enhanced needs of task coordination, distributed cognition, and permanent information flows [19, 23]. Thirdly, it has been suggested that ED providers need to align potential debilitating effects of multitasking in terms of omissions or performance decrements with potential benefits such as efficiency and performance gains [3, 4, 11, 12]. However, respective empirical and real-world investigations into the complex and potentially ambiguous consequences of multitasking for ED physicians’ experiences are missing [10, 15].

Our considerations were informed by models and concepts originating from cognitive sciences and multitasking research which postulate interrelations of task switching behaviors with mental workload, cognitive and stress outcomes [10, 24–26]. Respective models presume potential tradeoffs of benefits and costs in the process of task switching and associated sequelae for human performance and stress responses [19, 25, 27–29]. To this end, we anticipated cause-effect-relationships between multitasking behaviors and physicians’ responses in terms of increased stress and mitigated situation awareness [26]. Specifically, our investigation aimed to:

1. determine the prevalence of multitasking among ED physicians; and to
2. investigate associations between multitasking and provider-perceived outcomes, with specifically analyzing associations between multitasking and work stress (study 1) as well as between multitasking and situation awareness (study 2).

## Methods

### Design

We established a multi-method and multi-stage approach that combined standardized observations, concurrent physician reports on work stress and situation awareness, and ED registry data. We established two consecutive studies, i.e., study 1 and study 2, in the course of a larger quality improvement project in the respective ED. [30] Study 1 was conducted between November 2015 and January 2016, study 2 between November 2016 and January 2017. The Ethics Committee of the Medical Faculty, Munich University, approved the study (Nr. 327–15). All physicians received written and verbal information prior to data collection. Participation was voluntary and consent was obtained. Data collection, processing, and storage were conducted in accordance with General Data Protection Regulations.

### Study setting

Our study was conducted in the ED of a major metropolitan, academic medical center in Southern Germany. This interdisciplinary, 24 h ED has about 84.000 yearly visits. Regarding size, patient census, work organization, staffing, and technological provisions, it is one of the largest EDs in Germany. Staff include specialists from trauma surgery, internal medicine, neurology, as well as further specialists on call. The ED consists of three treatment areas according to patient’s chief complaints: examination and treatment rooms for (1) medical (mainly internal, neurological), as well as (2) trauma patients, and (3) one short-term observation unit.

### Data collection procedure

Observation sessions were randomly assigned across ED treatment areas based on a randomization scheme developed prior to study start. On days of data collection, trained observers shadowed ED physicians during 90-min sessions. Eligible physicians were specialists as well as residents. A stratified sampling procedure was deployed to allocate observations randomly across ED units and time of the day (mornings, afternoons). Three expert observers with a professional background in healthcare and human factors shadowed ED staff for 90-min sessions.

### Observations of ED physician multitasking

In each observation session, observers coded physician activities with an established tool that includes eleven activity categories and that is further suitable to determine multitasking [9, 31, 32].

Multitasking was coded if two activities were observed to be evidently carried out in a timely concurrent manner [6, 31]. The key criterion for the observer was an obvious, directly observable overlay of concurrent activities to code a multitasking incident, e.g., documenting while talking to a colleague or, talking on the phone while simultaneously conducting a diagnostic procedure on a patient [5]. Start and end time of multitasking episodes were recorded. We finally aggregated the proportion of time spent in multitasking (in %) for each individual observation session.

First, we established reliability of our observational tool for multitasking. Prior to both studies, we carried out non-systematic observation sessions to test the feasibility of the tool. Additionally, we discussed inconsistencies between observers. Then, we deployed systematic pairwise observations for testing and establishing inter-observer agreement. For study 1, we obtained an agreement of Gwet’s AC1 = .72 (95%CI .67,.76; 15 pairwise observations). For study 2, we obtained an Gwet’s AC1 = 0.80 (95%CI 0.76,.85; 8 pairwise observations). Both results indicate good inter-observer agreement and confirm the tool’s reliability [33].

### Physician-reported outcomes: stress and situation awareness

In both studies, observed ED physicians filled out a short self-assessment survey after each observation session concerning the following outcomes:

- *Perceived multitasking (studies 1 and 2)*: One question examined the perceived frequency of multitasking demands during the observation session. We used a question from an established work analysis tool [34]: ‘During the past observation, how often did you have to work on tasks simultaneously?’. The answers were provided on a visual analogue scale from 0 = ‘few’ to 10 = ‘very frequent’.
- *Stress at work (study 1)*: We utilized the stress scale of the State-Trait-Anxiety-Inventory-6 (STAI-6) that quantifies cognitive, emotional, and physical aspects of work stress [35]. It consists of six statements with adjectives, e.g., describing feeling calm (reversely coded), tense, or upset. The answer scale ranges from 1=‘no, not at all’ to 4 = ‘yes, completely’. Scale’s internal consistency was Cronbach’s alpha = .89.
- *Situation awareness (study 2)*: We used a short scale that operationalizes Endsley’s widely adopted model of SA composed of perception, comprehension, and projection [36–38]. It consisted of three questions: ‘I was able to perceive important information concerning my work (e.g., concerning patients, workflow)’ (perception); ‘I was able to understand important information concerning my work (e.g., concerning patients, workflow)’ (comprehension); ‘I was able to project developments of my work’ (projection). ED physicians answered on a visual scale ranging from to 0=‘very low’ to 10=‘very high’. Cronbach’s Alpha was = .81.

### Additional operational measures

To account for ED patient census and physicians’ workload at the time of observations, we obtained additional information on ED staffing (study 1: count of ED staff on duty during shift of observation; study 2: number of physicians on duty), patient load (count of ED patients admitted during day of observation), and patient acuity (ratio of patients triaged with ESI-levels 1 and 2 to overall patients being evaluated). Data were retrieved from ED’s registry data and the hospital information system.

### Statistical analyses

We first matched all observational and self-report data at the session level. Additionally, respective ED operational data was included for each session. First, we determined mean proportions of time spent in multitasking to total observation time (research question 1). Then, we calculated correlation analyses to identify bivariate associations between ED operational, observational, and self-report metrics (research question 2. Afterwards, we employed multivariate linear regression analyses to determine associations between observed multitasking and physicians’ self-reports after controlling for ED operational confounders. SPSS 24.0 was used for all statistical analyses (IBM Inc., Chicago).

## Results

In study 1, we matched data for 28 observation sessions and respective ED physicians’ self-evaluations with an overall observation length of 43.1 h. For study 2, we analyzed 31 observation sessions of overall 46.3 h.

### Observational study 1: multitasking and stress

We determined an overall proportion of multitasking activities of 18.74% among observed ED physicians (SD = 12.93%; Range min-max .95–54.08%). Mean duration of individual multitasking episodes was 1 min and 28 s (SD = 32 s).

Mean physician-rated multitasking during observation sessions was M = 4.60 (SD = 2.99) and perceived stress was M = 1.79 (SD = .54). The association between the count of ED staff on duty and physician-perceived multitasking almost reached significance (*r* = .37, *p* = .051) whereas the number of admitted ED patients was significantly associated with physicians’ experiences of multitasking (*r* = .45, *p* = .02). Both parameters were not associated with perceived stress (ED staff: *r* = .30, *p* = .12; ED patients: *r* = .03, *p* = .87). There were no relationships between the share of high acuity patients and perceived multitasking (*r* = .16, *p* = .41) nor provider stress (*r* = −.01, *p* = .96).

After controlling for the proportion of high acuity patients, number of admitted patients, and ED staffing, we identified a significant association between expert-observed multitasking activities and physician-reported multitasking frequency (Beta = .08, 95%CI .01–.16; standardized β = .36; *p* = .03). We furthermore found a significant relationship between expert-observed multitasking and physician-reported stress, such that higher multitasking was related to higher stress evaluations by physicians (B = .02, 95%CI .005–.03; β = .48, *p* = .01); cf. Table 1.

**Table 1:** Associations of multitasking and ED physicians’ stress and situation awareness

	Provider outcomes			
	Stress (study 1)		Situation awareness (study 2)	
	B [95% CI]	p	B [95% CI]	p
*ED workload data*				
Share of high acuity patients	.00 [−.09, .09]	.97	**.16 [.02, .30]**	**.02**
Count of patients in ED care	−.03 [−.13, .06]	.50	−.01 [−.02, .01]	.47
Count of ED staff present	**.07 [.00, .13]**	**.04**	.27 [−.05, .58]	.09
*ED physicians’ multitasking*				
Observed % of multitasking	**.02 [.00, .03]**	**.01**	**.08 [.02, .14]**	**.009**

Note: B non-standardized regression coefficient; p Significance, bold if *p* < .05; intercept not depicted; study 1 *n* = 28 observation sessions; study 2 *n* = 31

### Observational study 2: multitasking and situation awareness

We found an aggregated share of multitasking of 13.04% (SD = 9.37, Range 2.71–42.90%) with a mean duration of individual multitasking episodes of 1 min and 21 s (SD = 26 s).

Mean physician-rated multitasking was M = 4.42 (SD = 2.51). Concerning associations of perceived frequency of multitasking with ED staffing (i.e., physicians on duty) and patient numbers, we obtained one statistically significant relationship: A higher share of high acuity patients was related to lower levels of physician-reported multitasking (*r* = −.40, *p* = .03), yet not related to observed frequency of multitasking (*r* = −.21, *p* = .25). There were no significant associations of both multitasking measures with the number of ED physicians being present as well with the number of admitted patients.

Mean physician situation awareness was M = 6.94 (SD = 1.66). The three facets of situation awareness were perceived differently with higher ratings for perception of critical information (SA-item 1: M = 7.44; SD = 1.65) and comprehension of important information (SA-item 2: M = 7.63; SD = 1.62) compared to lower ratings for projection of developments (SA-item 3: M = 5.76; SD = 2.43).

Finally, we tested for uni- and multivariate influences of multitasking on ED physicians’ perceived situational awareness (cf. Table 1). After controlling for ED staffing and workload data, we identified a significant association of multitasking with perceived situation awareness: higher proportions of concurrent activities were associated with increased ratings of SA (B = .08, 95% CI .02, .14; β = .47, *p* < .01; cf., Table 1).

## Discussion

Multitasking is conceived as inherent and inevitable to ED work. However, respective research into the sequelae of ED physicians’ multitasking behaviors is limited, particularly in real-world ED settings. Our multi-stage and multi-source approach with two consecutive studies revealed that frequent multitasking was associated with higher work stress and increased situation awareness.

Our first aim was to determine the prevalence of multitasking among ED physicians. We obtained a multitasking prevalence between 13% (study 2) and 18.7% (study 1) of the observed time. This corroborates previous investigations that ED physicians multitask during a substantial amount of their work time [3]. Moreover, our numbers are in a similar range of previous studies among ED physicians in other ED settings [6, 8, 16]. EDs are dynamic work systems that are characterized by varying patient loads requiring ED staff to attend intermittently to multiple patient care responsibilities. Hence, there is a persistent demand to carry out tasks simultaneously to effectively cope with workload [5, 39]. Accordingly, we obtained significant associations between expert-observed and physicians’ self-perceived multitasking which strengthens the internal and concurrent validity of our findings.

Our second aim was to determine associations between multitasking and ED physician outcomes with particular focus on work stress and situation awareness. To the best of our knowledge, this is the first naturalistic study that empirically scrutinized sequelae of multitasking for ED provider experiences. After controlling for ED workload, we identified significant relationships between observed multitasking and physicians’ self-rated stress and situation awareness. Frequent multitasking behaviors were associated with higher stress: physicians seem to compensate multiple task demands through enhanced stress responses triggered through enhanced efforts and increased energetic expenditures [40]. Our results resonate with previous findings showing that ED provider’s multitasking was associated with increased mental workload [31]. However, due to mounting workload, stress during ED work might also be part of a feedback system with positive as well as negative effects on current performance and subsequent adjustments in ED operations [41, 42].

Against our assumptions, we observed that ED physicians who were frequently engaged in multitasking reported higher situation awareness. This result deserves careful consideration. Post-hoc we suggest two potential justifications for this finding: First, situation awareness is a key cognitive resource for ED physicians for effective performance in dynamic and complex care situations [43, 44]. Hence, multitasking might be an effective behavioral strategy to maintain oversight and attention to multiple concurrent streams of patient care [3]. Concurrent task performance may help providers to keep track of alerts concerning acuity and patient needs, proactive management of space, creation of opportunities for following patients, prioritization, and anticipation of tasks [29, 45, 46]. Since ED work requires identification and interpretation of situation and patient cues from multiple, often dynamically changing data streams and sources, synchronous allocation of attention and behaviors toward multiple task demands may spur providers’ abilities to keep track with various care processes [18, 20, 22, 43, 46]. A previous investigation into ED physicians’ decision-making revealed that particularly notions of priority were essential for task-scheduling decisions [47]. Consequently, if ED physicians tend to attend longer to high-priority interruptions, eventually, this may trigger more multitasking episodes [48].

Our second post-hoc interpretation pertains to the role of subjective appraisals of multitasking. Previous research proposed that people who engage in multiple activities through rapid task switching mentally construct this as multitasking with positive effects for performance outcomes, what has been proposed as ‘illusion of multitasking’ [49]. Although individual’s perception of multitasking is malleable, mere perceptions of engaging in two activities contributes to increased experiences of arousal and performance [49, 50]. We conjecture that physicians aim to master their work as adept multitaskers [15]. Previous research suggests that provider multitasking behaviors can be conceived as an optimization strategy to compensate for high clinical workload, insufficient work design, and suboptimal organization [5, 45]. Along this line, multitasking behaviors are a preferred strategy to master high workloads in fast-paced, time-constraint care environments [51]. Eventually, multitasking stipulates physicians’ experiences of mastery, self-confidence, and appraisal of performance [5, 15]. Multitasking may thus foster interpretation of situation cues from multiple, dynamically changing data streams, i.e., such as various information that can be critical or irrelevant; adaption to evolving situations; and keeping track of and utilizing special elements of knowledge [23, 43]. Since we observed particularly during phases of low or medium cognitive load and everyday ED care, our findings may pertain to times of routine work that allow for efficient task switching under opportune moments [24, 26]. Available literature on ED stress and SA has been focused on high-demanding emergency care task with immense efforts that overburden provider cognition and mitigate situation awareness, i.e., resuscitation, overcrowding [18, 41, 42]. Moreover, laboratory studies suggest that medium levels of discretionary task switching may spur productivity, nonetheless, at the cost of accuracy [27]. With regard to ED care, future studies may test for non-linear relationships between multitasking demands and outcomes, i.e., an inverted-U curve between multitasking and ED performance or quality outcomes [52]. To this end, our findings need to be replicated in studies across various ED settings as well as warrant further investigation concerning the role of medium- and high-complexity tasks and workload. Particularly, to test if multitasking might facilitate key aspects of physician’s situation awareness that draw upon cognitive and information resources of ED’s care system.

Altogether, our tentative findings challenge previous assumptions concerning the role of multitasking in ED care. Our results suggest that multitasking behaviors may come at the cost of subjective stress through overtaxing provider cognitive systems and overburdened psychological efforts, hence, with potential benefits resulting in increased experiences of situation awareness and alertness.

### Limitations

Various limitations apply to our study. Although we sought to establish a prospective assessment, our analyses limit inferences concerning causality. We presumed cause-effect relationships of multitasking behaviors with particular provider outcomes, i.e., stress and outcomes, and established consecutive and temporally independent measures of observations and provider appraisals. Yet, our design does not allow unequivocal inferences concerning the actual directions of the identified effects. As discussed above, finer grained studies are necessary to rule out potential alternative explanations, e.g., experiences of high situation awareness entice ED physicians into adopting more multitasking behaviors. Our findings stem from real-world ED care work and may feed future simulation or lab-based studies that allow a deeper understanding of the actual consequences of provider multitasking.

Both studies included convenience sampling approaches (i.e., selection of ED professionals being present at the randomly chosen time and location) and were restricted in sample size as well as response volumes (i.e., limited number of expert observations and post-hoc surveys within each study, respectively). Although we sought to combine various sources of data to limit bias and achieve a comprehensive assessment of provider multitasking, we acknowledge that our observational measures do not convey the full complexity of ED’s operational and sociotechnical care system [20, 21]. The external validity of our findings should be considered carefully. Subsequent investigations should draw upon multi-center trials that encompass varying complexities and dynamics of ED work settings across different hospital environments and national settings [53]. To strengthen internal validity, we merely assessed day shifts and mostly routine ED care demands. This limits inferences concerning night shift work or other times of irregular demands that may spur multitasking demands.

We deployed well-established tools to identify multitasking activities and associated provider outcomes. Yet, our definition and operationalization of multitasking activities deserves careful interpretation since it draws upon a simultaneous dual-task-performance notion [10]. Although we observed activities that were carried out in a timely concurrent manner, humans actually switch frequently between simultaneous task demands, i.e., intermittingly allocating attention and mental resources to both tasks in rapid cycles, also considered as interleaved multitasking or task switching which might not be observable [10, 24, 28]. Future investigations should also seek to scrutinize concurrent task combinations of complex or less complex activities and modalities, i.e., feasibility of concordant vs. discordant activities [10]. Moreover, we made no difference concerning voluntary (or internally prompted) compared to externally prompted multitasking behaviors what might incur with different implications concerning cognitive load, time for task completion, and likelihood of errors [10, 26]. Lastly, although high stress and mitigated situation awareness among healthcare professionals contribute to adverse outcomes, we cannot infer on subsequent adverse performance detriments as well as inferior patient outcomes, e.g., medication administration errors or flawed decisions [2, 12, 54, 55]. Lastly, we used a pragmatic measure of SA that relied on providers’ self-report with increased likelihood of subjective bias. Other conceptions and measures of SA have been introduced such as situated SA or sense making [36].

### Implications for ED practice and research

Multitasking behaviors are an almost fundamental part of ED physicians’ daily work routines. Our studies corroborate that ED physicians deal with multiple demands and need to coordinate tasks across various streams of patient care and multiple providers [12, 29, 31, 45]. Future studies are necessary that further scrutinize the complexities and dynamic characteristics of ED work, cognitive, and care systems [20, 21]. Further research into the role and potential benefits of multitasking in dynamic acute care is needed [3, 7]. Since multitasking tactics might be perceived as unavoidable in busy ED environments, it remains unclear how in practice patient care is compromised [7]. Among Australian ED physicians, multitasking was related to prescribing errors [55]. Notwithstanding, the often ascertained association of multitasking behaviors and errors is inconsistent and lacks substantial empirical evidence [10]. With respect to the role of provider experience or tenure that were not assessed here, further studies should test if senior physicians are more skilled in (re) engaging in multiple tasks with particular focus on performance outcomes, e.g., diagnostic accuracy [56]. Since ED professionals frequently operate under overload and competing demands, we need studies that evaluate tradeoffs between attributes of different tasks, associated switching tendencies, and cognitive outcomes [27, 57]. In-depth investigations are further necessary to elicit task complexities and deconstruction of task behaviors around interruptive events and episodes in ED care, i.e., ED physicians’ behavioral tendency for task switches to interruptive events while performing critical tasks such as ECG interpretation [11, 58].

Scholarly efforts are also necessary to investigate to what extent contemporary clinical settings with their dynamic workload and multiple timely demands stimulate clinicians’ preference to carry out tasks simultaneously, i.e., polychronicity as a behavioral response [55]. Although multitasking is considered a key skill for health care professionals, systematic training and formal developmental approaches that address task switching behaviors with respect to the possible negative effects of multitasking are still in their early stages. In ED practice, several avenues to address and teach the benefits and pitfalls of multitasking are feasible at different system levels: this may include on an individual level deliberate reflections on error-prone or inefficient work practices, accumulated experiences of safety-critical demands, and development of personal biases; on team-level, simulation approaches with post-hoc debriefings, senior supervision, crew-resource-management, delegation of duties, use of decision support tools; on an organizational level, provision of adequate resources, role-modeling by superiors, and implementation of technological support systems [3, 10, 12, 13, 55, 59]. Since multitasking is unavoidable in acute medical work, comprehensive and system-wide approaches are necessary that are cognizant of the complex tradeoffs of multitasking behaviors for safe management of multiple and often competing care demands.

## Conclusions

Our two empirical investigations revealed that multitasking behaviors were associated with increased stress as well as increased situation awareness among ED physicians. Results suggest that a nuanced understanding concerning the debilitating nature of multitasking in everyday emergency care work is necessary with particular focus on potential benefits or decrements for provider cognition and functioning. Moreover, further investigation into the factors that contribute to multitasking and potential errors in the ED is warranted.

## Supplementary Information

**Additional file 1: Table S1.** Uni- and multivariate associations of multitasking and ED provider outcomes stress and situation awareness.

## Data Availability

Anonymized data can be obtained from corresponding author on reasonable request.

## Abbreviations

CI: Confidence interval
ED: Emergency department
ESI: Emergency Severity Index
SA: Situation awareness
