# Supplementary material for: Multitasking behaviors and provider outcomes in emergency department physicians: two consecutive, observational and multi-source studies
Source: Scand J Trauma Resusc Emerg Med. 2021 Jan 7;29:14. doi: 10.1186/s13049-020-00824-8 (PMC7792086; doi:10.1186/s13049-020-00824-8)
Supplement: Supplementary file 1 — Additional file 1: Table S1. Uni- and multivariate associations of multitasking and ED provider outcomes stress and situation awareness. [file 13049_2020_824_MOESM1_ESM.docx]

**Supplementary Material**

Table S1: Uni- and multivariate associations of multitasking and ED physician outcomes stress and situation awareness

|  |  |  | **Provider outcomes** | | | | | | | | |
| --- | --- | --- | --- | --- | --- | --- | --- | --- | --- | --- | --- |
|  |  |  | **Stress** (study 1) | | | |  | **Situation awareness** (study 2) | | | |
|  |  |  | crude |  | adjusted |  |  | crude | | adjusted | |
|  | |  | B [95% CI] | p | B [95% CI] | p |  | B[95% CI] | p | B [95% CI] | p |
| *ED Workload Data* | |  |  |  |  |  |  |  |  |  |  |
|  | Share of high acuity patients |  | -.002 [-.10, .10] | .96 | .00 [-.09, .09] | .97 |  | .13 [-.02, .28] | .08 | **.16 [.02, .30]** | **.02** |
|  | Count of patients in ED care |  | .01 [-.09, .11] | .87 | -.03 [-.13, .06] | .50 |  | -.01 [-.03, .01] | .50 | -.01 [-.02, .01] | .47 |
|  | Count of ED staff present |  | .05 [-.01, .11] | .12 | **.07 [.00, .13]** | **.04** |  | .22 [-.13, .58] | .21 | .27 [-.05, .58] | .09 |
| *ED physicians’ Multitasking* | |  |  |  |  |  |  |  |  |  |  |
|  | observed % of multitasking |  | **.02 [.002, .03]** | **.03** | **.02 [.00, .03]** | **.01** |  | .06 [.00, .12] | .36 | **.08 [.02, .14]** | **.009** |

Note: B non-standardized regression coefficient; p Significance, bold if p < .05; intercept not depicted; study 1 n=28 observation sessions; study 2 n=31 observation sessions.
